# Delayed Bleeding After Retrograde Intrarenal Surgery: A Rare Complication in Ectopic Pelvic Kidney

**DOI:** 10.7759/cureus.23672

**Published:** 2022-03-31

**Authors:** Ernesto Reggio, Diego M Souza, Roberto G Junqueira, Marcelo J Sette, Carlos S Bellucci

**Affiliations:** 1 Urology, Uroclínica de Joinville, Joinville, BRA; 2 Cirurgia, Hospital Dona Helena, Joinville, BRA; 3 Cirurgia, Universidade Univille, Joinville, BRA; 4 Cirurgia, Uroclínica de Joinville, Joinville, BRA

**Keywords:** superselective embolization, retrograde intrarenal surgery, surgical complication, nephrolithiasis, pelvic ectopic kidney

## Abstract

Anatomical variations in the pelvic ectopic kidney (PEK) present many challenges to stone treatment. Retrograde intrarenal surgery (RIRS) has emerged as the treatment of choice for small to medium stones. We present a case of delayed hemorrhage due to an arteriocaliceal fistula. A 57-year-old man with a 12 mm middle calyx stone was subjected to uneventful RIRS, despite a high grade of scope deflection. Recovery was unremarkable until 37 days after surgery when the patient started recurrent hematuria and clot retention. Renal angiography revealed a bleeding vessel from an arteriocaliceal fistula. Superselective arterial embolization was successfully performed. Anomalous collecting system and vasculature can increase the risk of complications in PEKs. Massive bleeding from unusual arterial blood supply was effectively treated by angioembolization.

## Introduction

Anatomical variation in anomalous kidneys poses a potential drawback for endoscopic stone treatment. As retrograde intrarenal renal surgery (RIRS) has enhanced, most cases are successfully treated, despite troublesome access and fragments retrieval [1]. The treatment of stones in the pelvic ectopic kidney (PEK) demands flexible ureteroscopes with a high degree of flexion and deflection capabilities.

Vascular abnormalities are the rule for ectopic kidneys. Multiple vascular sources may be found and must be considered in the course of treatment [2].

Renal stone access may be achieved using prolonged and excessive stress applied to the deflection mechanism and maneuvers in the contorted collecting system, increasing the risk of vascular trauma.

## Case presentation

A 57-year-old male presented with mild recurrent pelvic pain. Clinical examination showed a lower abdominal tenderness. He had no significant medical history or drug administration. Radiological investigation with computed tomography showed a 12 mm middle calyceal stone within a right-side PEK.

Informed consent was obtained, and RIRS was carried out. Under general anesthesia, the patient was positioned in supine lithotomy positioning. Cystoscopy showed typical bladder anatomy with ureteral orifices normally located; a safety guidewire was placed via the ureteral orifice in the kidney. A ureteral catheter was inserted over the wire, and a retrograde ureteropyelography confirmed a PEK and a non-dilated urinary system. The guidewire was kept in the ureter, and Karl Storz 9-F semi-rigid ureteroscope was inserted to evaluate and passive dilate the distal ureter.

A flexor ureteral access sheath (ID: 10.7F/35 cm) was placed under fluoroscopic control with the distal extremity just below the ureteropelvic junction. We used a digital 9.2F reusable ureterorenal endoscope; excessive and prolonged deflections were required to access the stone. The irrigation method was a pressurized saline bag with a manually inflatable cuff; intrarenal pressure was not controlled.

**Figure 1:**
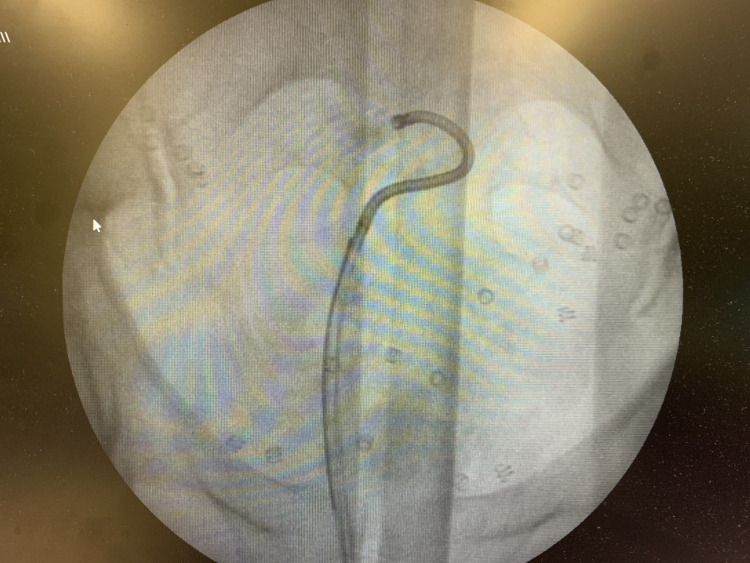
High-grade endoscope deflection.

Laser fragmentation was necessary to reduce the stone size and move it to a more favorable calyceal anatomy. The laser setting was 0.5J x 20Hz. Subsequently, lithotripsy was completed, and the fragments were retrieved using a nitinol frontal stone basket. There was no bleeding throughout the procedure; at the end of the surgery collecting system was checked for residual fragments, and no vascular trauma was noted, including the calyx where the stone was originally located. A Universa Soft Ureteral Stent (4.7F/22-32 cm; Cook Medical, Indiana, US) was placed, and the position was confirmed by fluoroscopy. The operative time was 49 minutes. The patient was discharged on the first postoperative day without hematuria, and the stent was removed 10 days later. Thirty-seven days after surgery, the patient presented at the ED complaining of gross hematuria without other symptoms. The CT scan was unremarkable, and on the following day, the patient was discharged home with clear urine. Four days later, he returned to the hospital with discomfort and difficulty passing the urine because of large clots. CT scan showed clots in the pelvic kidney collecting system and bladder. Renal angiography was performed, and a bleeding vessel from an arteriocaliceal fistula in the middle calyx, where the stone was originally located, was identified. The blood supply to this area was provided by the median sacral artery. Selective catheterization with a microcatheter was performed, and the lesion was occluded using microspheres. Complete resolution of bleeding was observed, and the patient was sent home with mild hematuria the next day.

**Figure 2:**
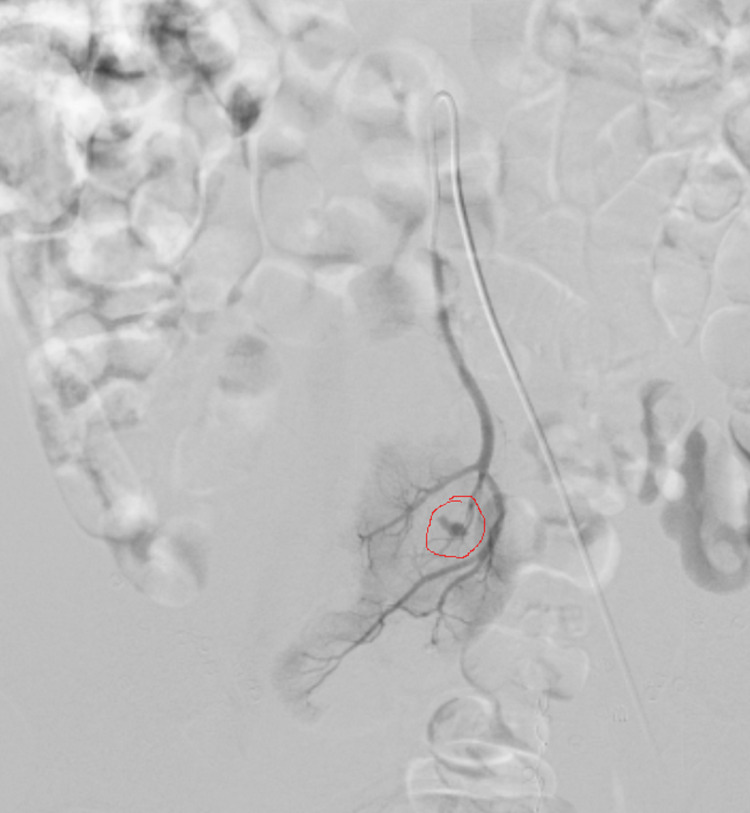
Ectopic pelvic kidney angiography shows arteriocaliceal fistula.

**Figure 3:**
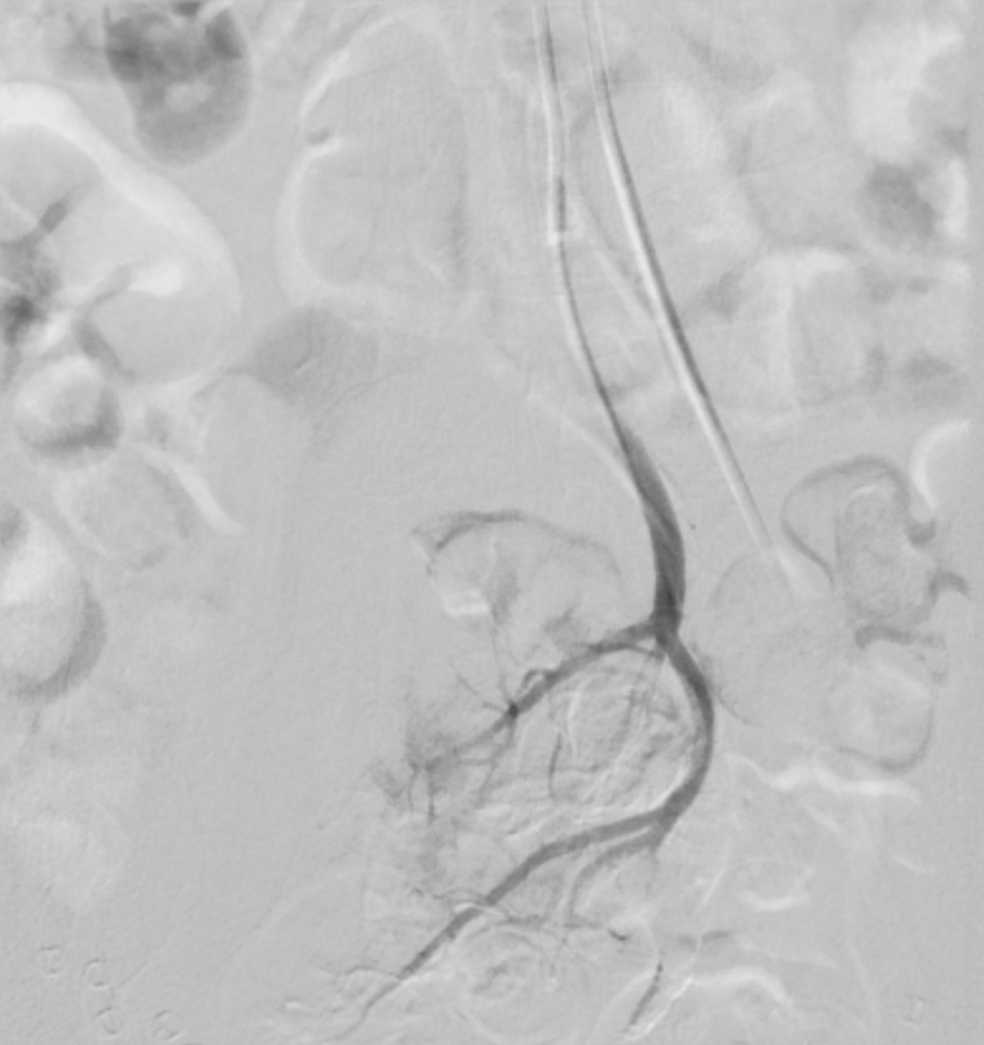
Arteriography shows successful selective embolization with complete bleeding control.

.

## Discussion

Despite PEK being habitually asymptomatic, patients are more likely to develop urolithiasis when compared to the normal population. In addition, shockwave lithotripsy within PEK is associated with lower success rates secondary to impaired stone clearance and limited stone localization and targeting [3]. Percutaneous nephrolithotomy (PCNL) has higher stone-free rates, especially for large renal calculi; however, it is more technically challenging due to the abnormal renal and vascular anatomy.

In this scenario, RIRS has been widely used and is recommended as the treatment of choice for small-to-medium-sized renal calculi. Recent advancements in laser lithotripsy and digital endoscopes have improved the stone-free rate (84.6%) [4]. Nevertheless, anatomic variations with multiple abnormalities demand expertise with unexpected findings as tortuous ureter makes the ureteral access sheath insertion difficult. Unsuccessfully treatments were associated with the impaired passage of fragments or inability to reach stones. Bozkurt OF et al. [5] investigated the outcomes of RIRS in 26 patients with PEK. Stone relocation and dusting lithotripsy were used. Although the treatment was successful in 84.7%, it failed in patients with unfavorable infundibulopelvic anatomy. In this case, we could easily insert the access sheath; however, a high grade of endoscope deflection was necessary to reach the stone. Moreover, laser fragmentation was necessary to reduce stone dimensions and move it to a more suitable calyx. Another potential risk was the high caliceal pressure during this surgical step since the calyx infundibulum was narrow. Those maneuvers might be traumatic and lead to late caliceal and vascular injury [6].

Late bleeding was a peculiar complication and, up to this point, unexplainable. At the end of the procedure, we double-checked the collecting system, and we could not find any injury. Delayed bleeding after PCNL has been well described [7] due to the rupture of pseudoaneurysm or arteriocaliceal fistula formed by a higher difference in blood pressure between an injured artery and an injured artery calyx. High irrigation pressure and excessive endoscope maneuvers might be the cause of the delayed hemorrhage. Moreover, complicated and highly variable vasculature must be kept in mind to avoid the risk of vascular injury.

## Conclusions

RIRS is the treatment of choice for small and medium-size PEK stones. Complications are atypical; however, care must be taken to reduce high pressure and more traumatic endoscopic maneuvers during the procedure. Late bleeding was successfully treated by superselective embolization.
